# Sonographic images of hepato-pancreatico-biliary and intestinal ascariasis: A pictorial review

**DOI:** 10.1007/s13244-015-0428-7

**Published:** 2015-09-16

**Authors:** Donboklang Lynser, Akash Handique, Chhunthang Daniala, Pranjal Phukan, Evarisalin Marbaniang

**Affiliations:** Department of Radiology and Imaging, Ganesh Das Hospital, Lawmali, Shillong, 793001 Meghalaya India; Department of Radiology and Imaging, North Eastern Indira Gandhi Regional Institute of Health and Medical Sciences, Mawdiangdiang, Shillong, 793018 Meghalaya India; Department of Pathology, North Eastern Indira Gandhi Regional Institute of Health and Medical Sciences, Mawdiangdiang, Shillong, 793018 Meghalaya India

**Keywords:** Ascariasis, Ultrasound, Acute abdomen, Cholecystitis, Pancreatitis

## Abstract

**Abstract:**

Despite advancement in the diagnosis and treatment of intestinal helminthiasis, ascariasis remains the most common cause of helminthic infections in the developing countries. Ultrasound offers a rapid, safe, and noninvasive approach to the diagnosis of intestinal ascariasis. Ultrasound is also the modality of choice for diagnosis of hepatobiliary ascariasis, which is relatively rare and is due to migration of intestinal worms through the papilla of Vater. We present an imaging spectrum of hepato-pancreatico-biliary and intestinal ascariasis.

***Main messages*:**

• *Ascariasis refer to infestation by the round worm ascaris lumbricoides*.

• *Ascaris eggs are excreted in faeces and are infective to humans*.

• *Eggs hatch to larva*, *travel to the lungs and mature to adult worms*.

• *Intestinal obstruction can be caused by multiple ascariasis forming bag of worms*.

Ascariasis is a common problem, with approximately one-fourth of the world population infected [1]. The majority of infections occur in the developing countries of Asia and Latin America [2]. Ascariasis refers to infestation by the roundworm Ascaris lumbricoides. Humans become infested after ingesting material contaminated with embryonated eggs from faeces of an infected individual. The eggs hatch to larvae in the small intestine under the influence of gastric secretions. The larvae then penetrate the intestinal mucosa and are transported haematogenously to the lungs. The worm then matures in the alveoli and then travels up the bronchi and trachea only to be swallowed again. Once in the intestine, they mature to adult worms. They then mate and produce eggs. The eggs are then excreted along with faeces and are infective to humans, thereby completing the cycle.

## Ultrasound imaging of intestinal ascariasis

**Fig. 1:**
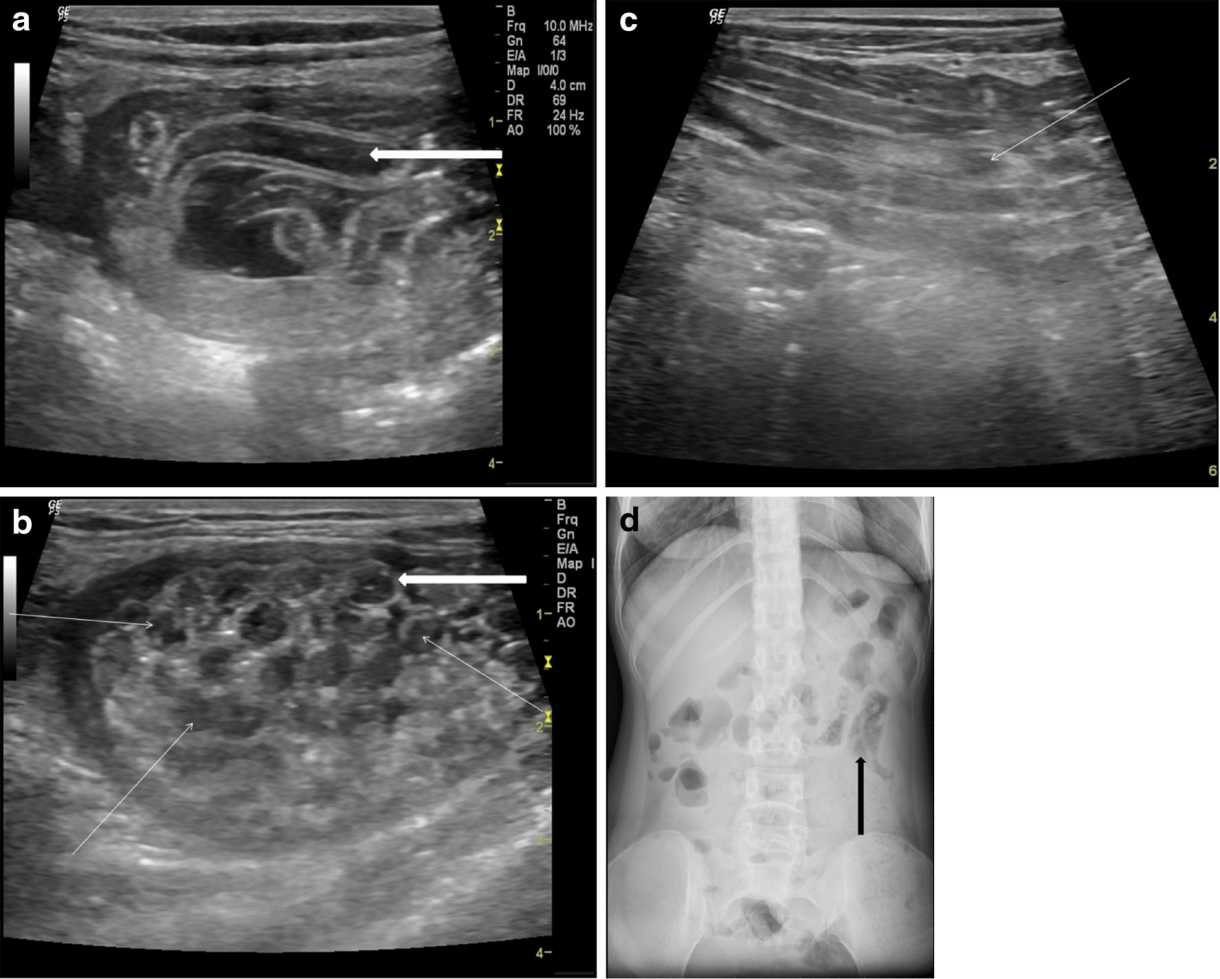
A 25-year-old female with acute abdomen and clinical signs of intestinal obstruction. (**a**) High resolution ultrasound using linear array transducer showing intestinal ascariasis, note distended fluid filled alimentary canal of the ascaris (*thick white arrow*), the “inner tube sign”, (**b**) same patient showing multiple ring like shadows (*thin white arrows*), some with target appearance (*thick white arrow*) on transverse section of the intestinal lumen indicating bag of worms, (**c**) “Bag of worms” in a long section (*thin white arrow*) and (**d**) PA erect X-ray abdomen of this patient showing tubular soft densities suggestive of ascariasis (*thick black arrow*)

Adult worms are seen as tubular structures outlined by intestinal fluid. The adult worm is seen as a large, curved echogenic strip with an inner, anechoic, longitudinal canal [3]. When we used a high-resolution linear (7–10 mhz) transducer (Logiq P5, GE Milwaukee US), in long section the worm appeared as four parallel lines separated by three anechoic bands (Fig. 1a). In cross section, it is round and sometimes appearing as a “target sign” [4]. The live worms are sometimes seen moving in the intestinal lumen. At times they are multiple forming a bag or cluster of worms, which can cause intestinal obstruction (Fig.1b&c). On rare occasions, the round worm can also be seen in a plain x-ray abdomen (Fig. 1d).

## Hepatobiliary ascariasis

Although no reliable data exists, hepatobiliary ascariasis is far less common compared to intestinal ascariasis alone. Patients with hepato-biliary ascariasis can present as biliary colic, acute cholecystitis, acute cholangitis, acute pancreatitis, and hepatic abscess. They may also present later as intrahepatic duct calculi due to recurrent biliary invasion [5]. Ascariasis in the biliary tract can have any of the following imaging features on sonography [6].

1. Inner-tube sign: The roundworm may be seen as a thick echoic stripe with a central, longitudinal anechoic tube (gastrointestinal tract of the worm) in a gall bladder or CBD.
2. Coil of worm in the gall bladder.
3. Strip sign: Thin non-shadowing strip without an inner tube in the CBD or gall bladder.
4. Spaghetti sign: Overlapping longitudinal interfaces in the main bile duct.
5. In our observation, we have found several linear calcified structures within the bile ducts that might be calcified dead worms, possibly representing remote hepatobiliary infestation.

## Common bile duct (CBD)

**Fig. 2:**
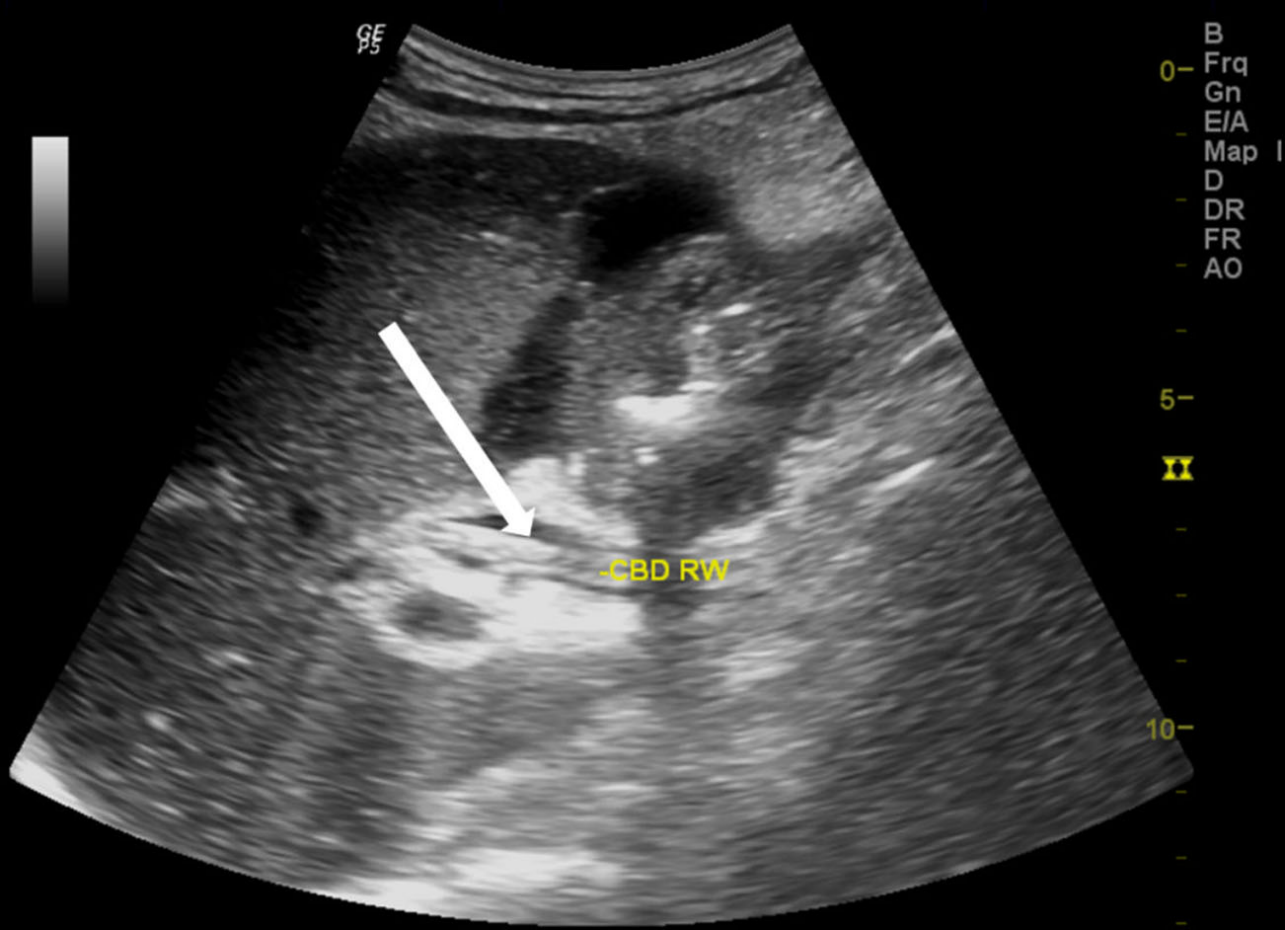
A patient with acute epigastrium, CBD showing double linear echogenic wall indicating ascariasis”strip sign” (*white arrow*)

**Fig. 3:**
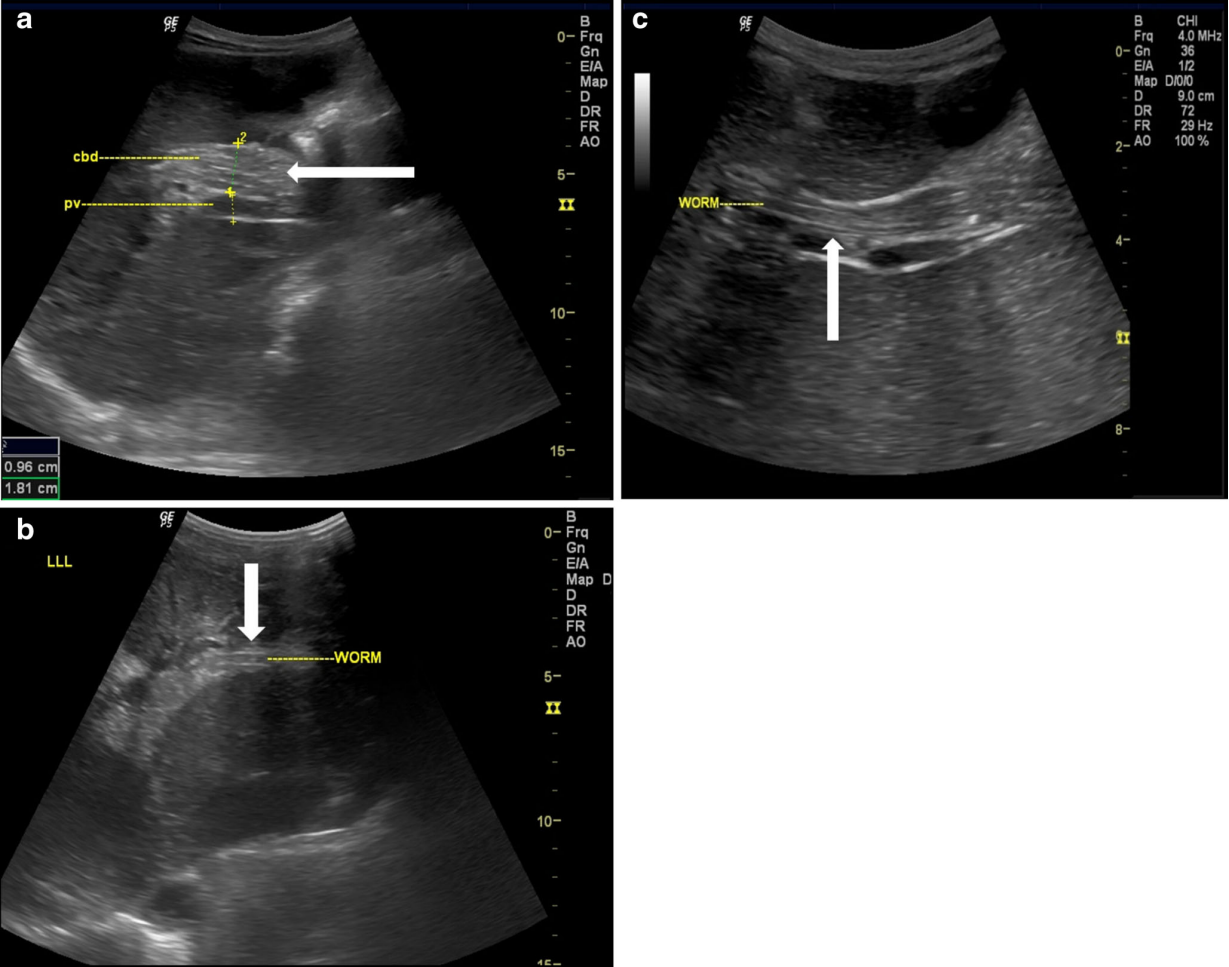
A 14-year-old female with acute biliary colic. (**a**) Multiple ascaris in CBD, the spaghetti sign, CBD is filled with multiple worms (*long thick white arrow*), (**b**) intra hepatic duct of the left lobe of liver showing a double linear echogenic wall indicating ascariasis (*white arrow*) and (**c**) following antihelminthic medication, sonography was repeated the next day showing reduction in the number of worms. Note double echogenic walls (*strip sign*) of CBD round worm (*white arrow*)

**Fig. 4:**
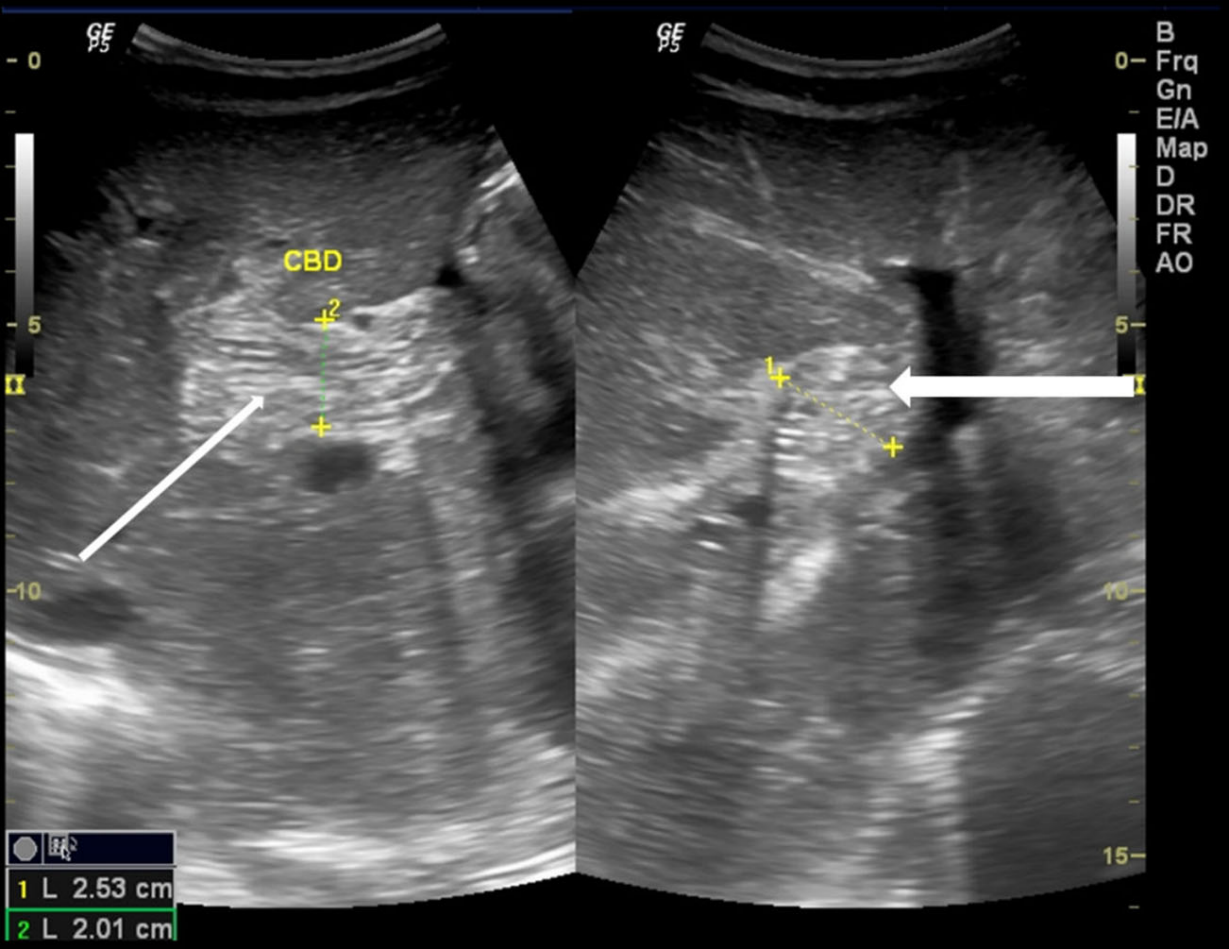
A 35-year-old male with acute abdomen showing pseudotumour-like shadows in the CBD (*thick white arrow*). Note that the spaghetti sign is seen in the long section (*thin white arrow*)

The worm in the CBD can be seen as single or multiple tubular, linear echogenic non-shadowing walls (strip sign) (Fig. 2). On rare occasions, too many worms inside the CBD can appear as multiple echogenic non-shadowing linear interfaces, giving a classical spaghetti sign (Fig. 3a ,b and c). CBD round worms sometimes has a pseudotumour appearance (Fig. 4). CBD and intrahepatic ducts can become very dilated; however, obstructive jaundice is exceedingly rare.

## Intrahepatic ducts

**Fig. 5:**
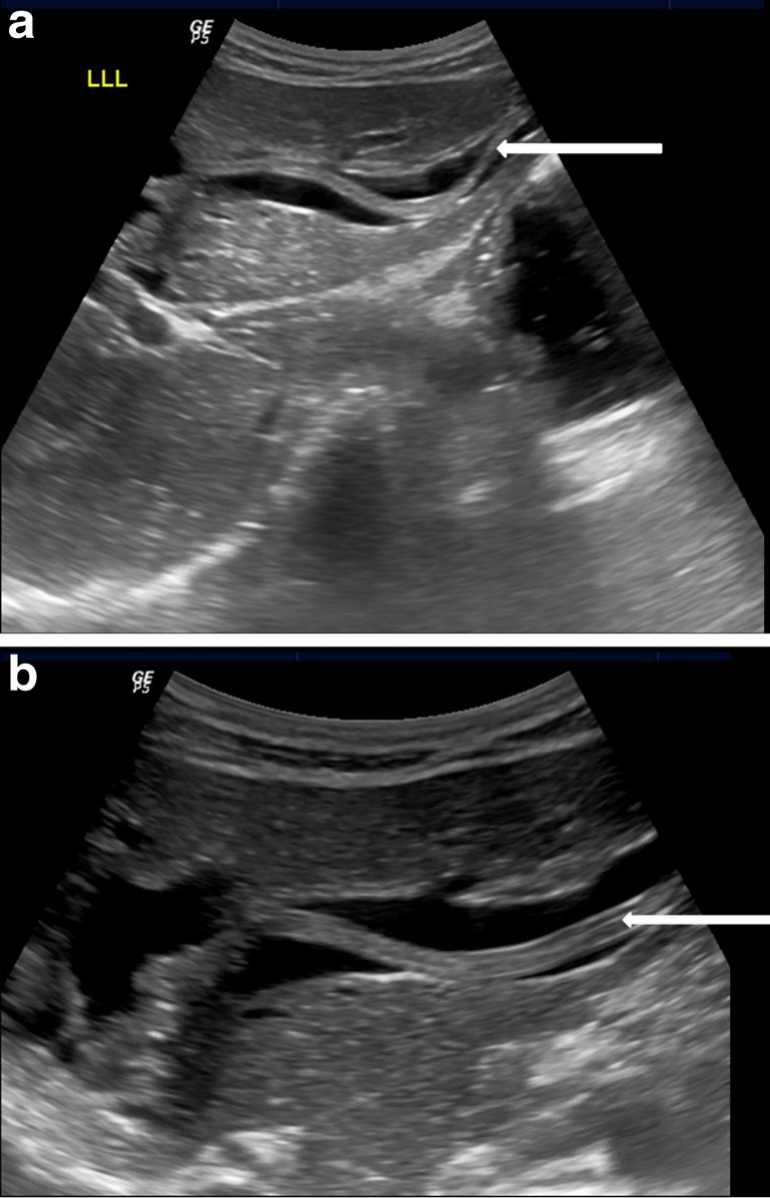
A 35-year-old female with acute pain in the right hypochondrium. (**a**) Ascaris seen in the left dilated intrahepatic duct (*white arrow*) and (**b**) magnified view of the ascaris in the dilated left intrahepatic duct, triple line seen (*white thick arrow*)

**Fig. 6:**
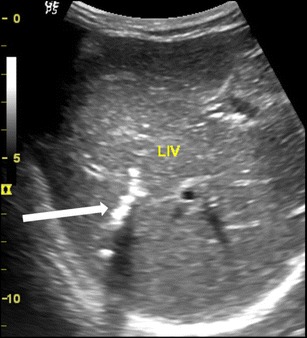
A 40-year-old female showing calcified shadow indicating calcified worms in the intrahepatic duct of the right lobe of liver (*thick white arrow*)

Ascaris in the intrahepatic ducts are less common than the common bile duct. They may be seen in either lobes of liver with intrahepatic biliary dilatation. They are seen as similar tubular structures inside the hepatic ducts with a “strip sign” (Fig. 5a). On rare occasion, a “triple line” can also be appreciated on magnified view or with the use of high resolution linear array transducer (Fig. 5b). Calcified worms in the intrahepatic duct are also occasionally seen in asymptomatic patients on routine sonography (Fig. 6).

## Gall bladder

**Fig. 7:**
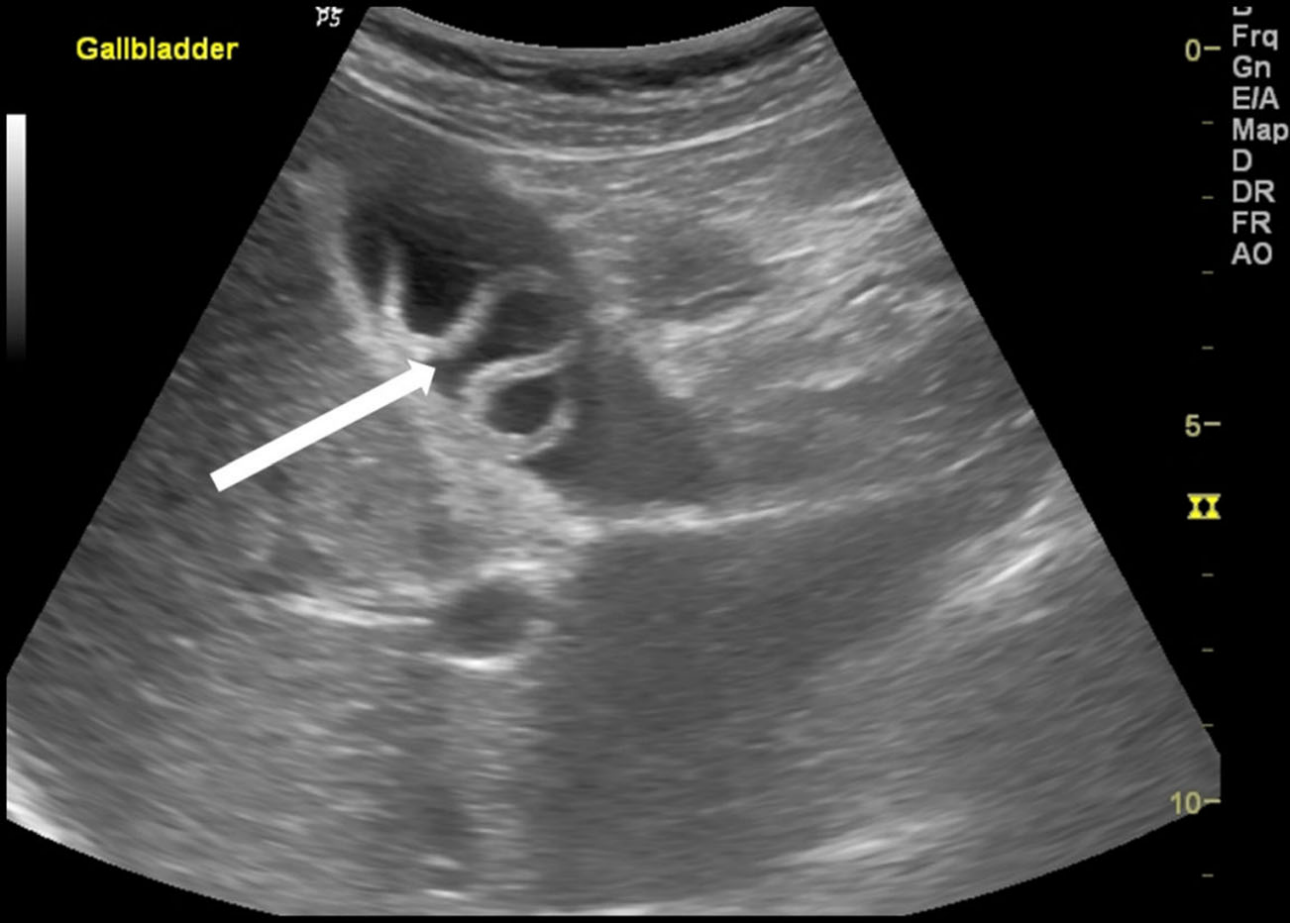
A 25-year-old female showing coil of ascariasis inside the gall bladder lumen (*white arrow*)

**Fig. 8:**
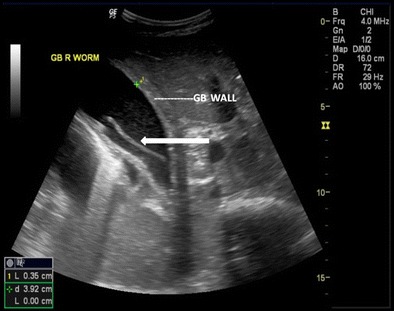
A 25-year-old male with acute right hypochondriac pain showing ascaris (*thick white arrow*) inside the gall bladder. Note GB wall thickening and luminal sludge indicating acute cholecystitis

Ascaris in the gallbladder is rare, constituting 2.1 % of hepatobiliary ascariasis [7]. This can be easily picked up on ultrasound. Inner tube may not be seen (Strip sign). Live worms may move within the gall bladder. The ascaris may be seen as a coil of worm in the gall bladder lumen (Fig. 7). The worm in the gall bladder may also present with cholecystitis (Fig. 8).

## Pancreatic duct

**Fig. 9:**
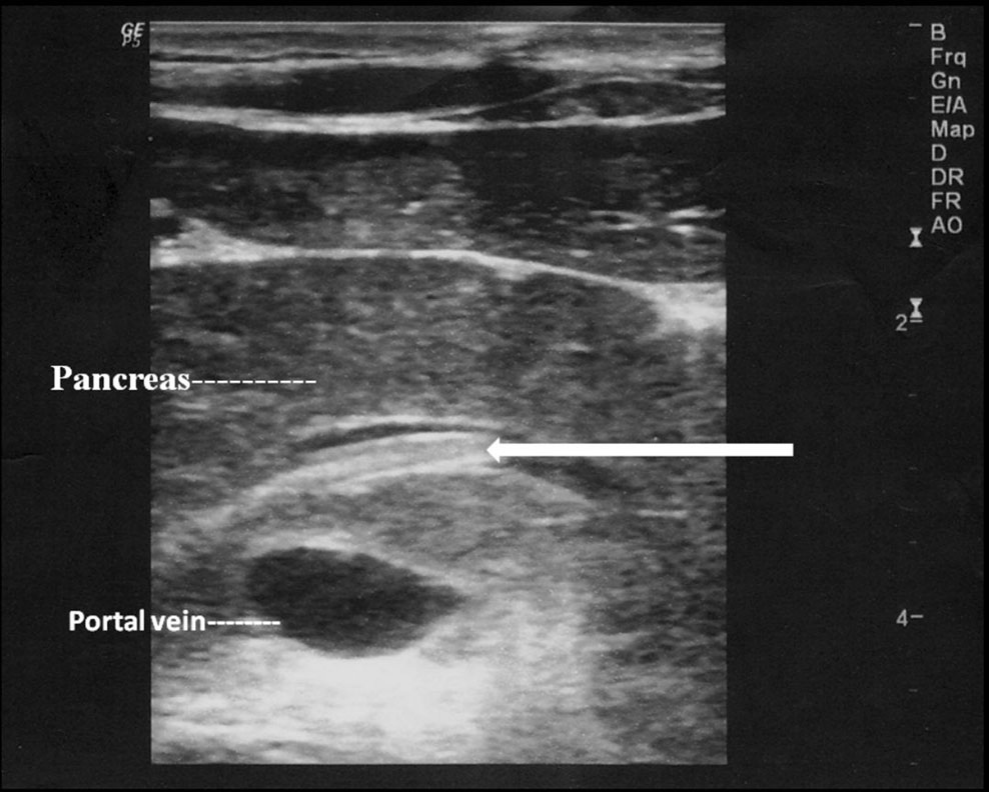
A 15-year-old male presented with acute epigastric pain and elevated pancreatic enzymes. High resolution ultrasound using linear array transducer shows ascaris with triple line within in the dilated main pancreatic duct (*long white arrow*) with pancreatitis

Rarely, ascaris in the main pancreatic duct can present with pancreatitis. The main pancreatic duct may be dilated, with “triple line sign”, “strip sign” or an “inner tube sign” and occasional pancreatic oedema (Fig. 9).

## Discussion

Multiple Ascaris Lumbricoides round worms can result in intestinal obstruction, or even cause volvulus with consequent necrosis of the involved bowel segment [8]. Magnetic resonance cholangio-pancreatography (MRCP) also has a role in the diagnosis of hepatobiliary ascariasis [9], and though other parasites can be involved in the biliary system [10], ultrasound with all its typical imaging features has a definite role in the diagnosis of hepato-pancreatico-biliary and intestinal ascariasis.

## Conclusion

In conclusion, clean hygiene prevents contamination of foods by ascaris eggs, therefore arresting the natural cycle. Sonography, on the other hand, is a noninvasive, cheap, rapid and safe imaging modality in hepato-pancreatico-biliary and intestinal ascariasis, especially in situations where other types of imaging are costly or inaccessible.
